# Trigger Features for Conveying Facial Expressions: The Role of Segmentation

**DOI:** 10.1177/2041669517737792

**Published:** 2017-11-21

**Authors:** Vilayanur S Ramachandran, Chaipat Chunharas, Michael Smythies

**Affiliations:** Center for Brain and Cognition, 8784 University of California, San Diego, La Jolla, CA, USA; Center for Brain and Cognition, 8784 University of California, San Diego, La Jolla, CA, USA; King Chulalongkorn Memorial Hospital, Chulalongkorn University, Thailand; Center for Brain and Cognition, 8784 University of California, San Diego, La Jolla, CA, USA

**Keywords:** face perception, segmentation, trigger feature, emotion

## Abstract

Primates are especially good at recognizing facial expression using two contrasting strategies—an individual diagnostic feature (e.g., raise eyebrows or lower mouth corner) versus a relationship between features. We report several novel experiments that demonstrate a profound role of grouping and segmentation—including stereo—on recognition of facial expressions.

The idea of *trigger features* is well known in ethology. Instead of responding to the *tout ensemble* or gestalt—as is usually the case—the organism responds to certain diagnostic features that circumvent the need for high-level processing. Subsequent activations of limbic structures results in rapid evocation of certain emotions and corresponding behavior. Baboons responding to purple red rumps of females in estrus (the mainstay of many nature films), seagull chicks pecking the red spot at the tip of a disembodied beak, and nightingales regurgitating food into the huge gaping mouths of grotesquely large cuckoo chicks are good examples. In humans, best examples of trigger features are perhaps facial expressions as demonstrated by face inversion effect (Thompson, 1980; other body parts like breasts may also qualify—male *homo sapiens* have a pronounced mammo-ocular reflex; Stuart Anstis, personal communication).

The trigger-like action of facial expressions can be seen in Figure 1(a), which shows that a wide range of expressions can be evoked using simple tilted lines, curves, and dots (e.g., compare a smiley face with a frown). Despite the fact that all details have been removed and the stimulus has been stripped to a bare minimum, it nonetheless evokes recognition of emotions almost as powerfully as real photos. Yet the mere presence of features is inadequate (e.g., Figure 1(b)), and the relationship between features is critical (Kosslyn et al., 1989). A more striking demo of this is shown in Figure 2, which is bistable. If the tilted eye brow line is assigned to the *left* circle, it becomes unmistakably imbued with anger, but if assimilated into the *right* circle, it triggers sadness. Even though the feature itself has not changed physically, it becomes imbued with the emotion that is ascribed to the whole face. An equally striking example of the powerful role of segmentation is shown in Figure 3. In Figure 3(a), the features within the circle are seen as mere fragments. But the addition of an occluding mustache (Figure 3(b)) results in amodal completion of the mouth behind a mustache, thereby conveying a smile.

**Figure 1.**
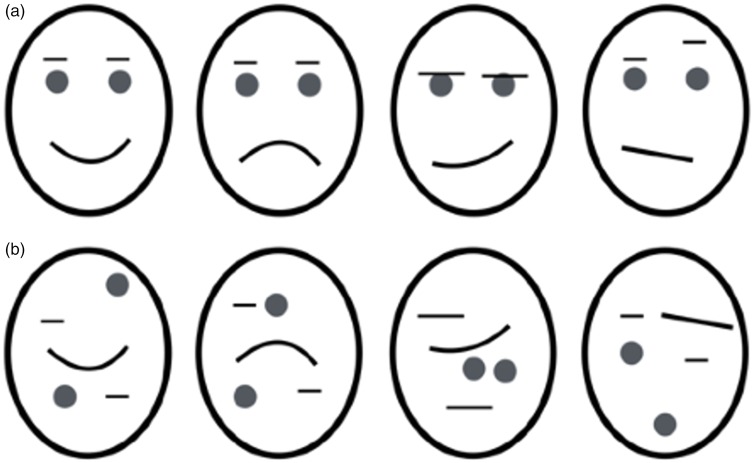
(a) A wide array of complex facial expressions can be conveyed by adjusting the locations and relative distances of simple lines and dots. (b) Illustration indicating that the mere presence of features is not enough, and the relationship between them is critical.

**Figure 2.**
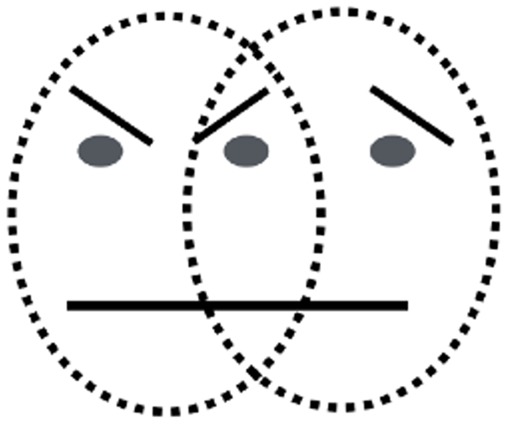
The features of overlapping zone of the two faces, when attributed to one of the two faces, give a distinct impression of belonging to that face. The features themselves do not change but become tainted with the emotion that is ascribed to the whole face.

**Figure 3.**
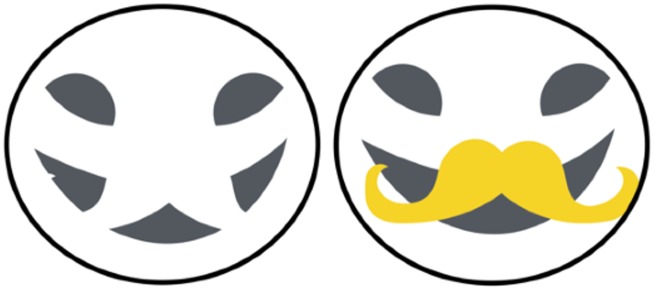
Two images demonstrate that perceptual continuity of the mouth behind the opaque mustache evokes a smile (b) whereas the same fragments fail to do so without mustache (a).

While exploring a wide range of stimuli, we discovered something quite odd, illustrated by comparing the two faces in Figure 4(a) and (b). Figure 4(a) clearly conveys a smile which is consistent with the *coarse coding hypothesis*: The mere presence of a curve with two tips pointing upward—in this case depicted by a banana—signal smile despite being recognized as a banana at a conscious cognitive level. The convexity bulging down contour is an ethological releasing signal. Yet this simple explanation is contradicted by Figure 4(b); the same banana placed at the correct location on a full color photo of a face does *not* evoke recognition of a smile—even though there is more information that it is a face than the mere circle and dots and eyebrow lines of Figure 4(a).

**Figure 4.**
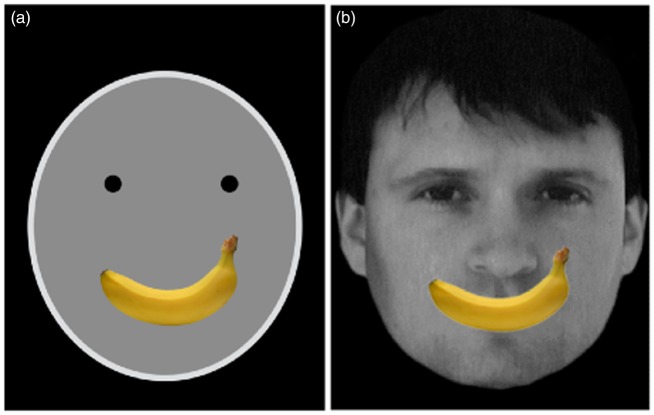
(a) A representation of a smiling face, depicted by two dots and a banana. (b) A photograph of a real person with a banana superimposed on the mouth. The result is a more convincing smile in (a) than in (b).

How does one interpret this result? Upon seeing the real face rich in detail, the neural network responding to the face is powerfully activated and is perhaps more demanding of equally strong confirmation from features conveying expressions—a banana will not do, and a mouth is needed. In the cartoon, on the other hand, nearly all the features are trimmed down to a bare minimum and therefore the need for distinctiveness of the one feature in question (mouth) is substantially relaxed—so even the fruit is effective despite complete lack of resemblance to lips.

We believe that these findings illustrate a new principle in perception. Imagine you wander into a completely new visual scene and it is very foggy—all objects are seen blurred. If there is a single road sign saying “MANCHESTER ROAD” seen in fragments partially hidden by fog, for example, “..AN..HEST..ER R..AD,” you are more willing to accept degraded information from the sign, lowering your perceptual threshold. But if there is no fog and all the other objects are clear, you would be reluctant to read it as “MANCHESTER ROAD.”

Figure 5 also illustrates this; a house with a banana mouth is paradoxically more effective as a face than a face photograph with a banana mouth! Again, what counts is the *consistency* in how indistinct or impoverished the different cues are, rather than the presence of a few diagnostic features. The house with the banana mouth is so unlikely an *accidental* juxtaposition that the brain is willing to accept that the only explanation is that it is a face. As Sherlock Holmes said “When you have ruled out the impossible, my dear Watson, whatever remains—however improbable—must be the correct answer” (Doyle, 2010).

**Figure 5.**
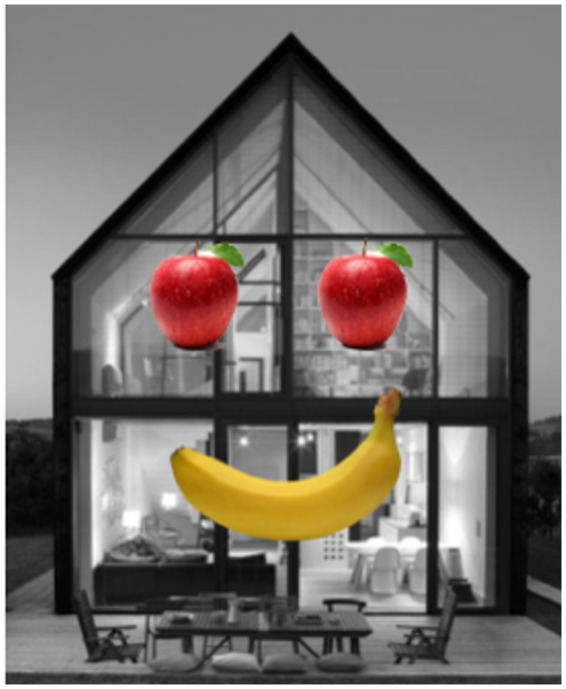
A house with a banana mouth evokes the appearance of smile.

As noted earlier, knowing which feature belongs to which object (which is constrained by image segmentation) is a necessary prelude to the recognition of emotional expression. To explore the role of segmentation further, we optically *grafted* a smiling real mouth on the photo of a face and simply put it in a different stereo plane floating out in front of the paper (Figure 6). Four subjects confirmed this loss of emotion. The smile goes away since the brain no longer groups the lips with the face.

**Figure 6.**
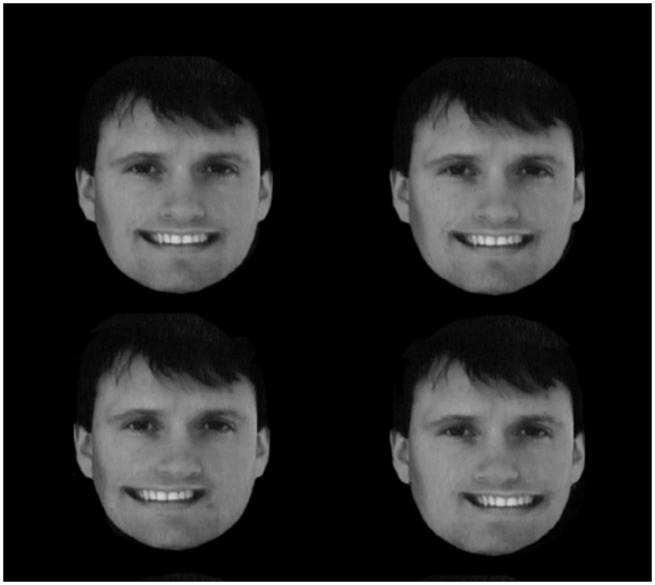
The mouths were placed such that when the images on the left and the right were fused, the mouth is in the same stereo plane (top row) or floating in front of the face (lower row). The lower face does not look like a smiling face compared to the upper face.

Taken collectively, our results together with those from Nakayama, Shimojo, and Silverman (1989) introduce a novel probe for understanding the manner in which the brain parses the visual image into objects—such as faces—and assigns different values to different features defining the object. Face detecting cells are abundant in fusiform gyrus (Kanwisher, McDermott, & Chun, 1997; Pinsk, DeSimone, Moore, Gross, & Kastner, 2005; Tsao, Freiwald, Tootell, & Livingstone, 2006) and our stimuli could be used to explore the underlying circuitry.
